# Direction‐averaged diffusion‐weighted MRI signal using different axisymmetric B‐tensor encoding schemes

**DOI:** 10.1002/mrm.28191

**Published:** 2020-02-21

**Authors:** Maryam Afzali, Santiago Aja‐Fernández, Derek K. Jones

**Affiliations:** ^1^ Cardiff University Brain Research Imaging Centre (CUBRIC), School of Psychology Cardiff University Cardiff United Kingdom; ^2^ Laboratorio de Procesado de Imagen, ETSI Telecomunicación Edificio de las Nuevas Tecnologías Universidad de Valladolid Valladolid Spain; ^3^ Mary MacKillop Institute for Health Research, Faculty of Health Sciences Australian Catholic University Melbourne VIC Australia

**Keywords:** B‐tensor encoding, diffusion‐weighted MRI, direction‐averaged diffusion signal, high b‐value, power‐law

## Abstract

**Purpose:**

It has been shown, theoretically and in vivo, that using the Stejskal‐Tanner pulsed‐gradient, or linear tensor encoding (LTE), and in tissue exhibiting a “stick‐like” diffusion geometry, the direction‐averaged diffusion‐weighted MRI signal at high b‐values ( $7000\;<\;b\;<\;10000\;s/{\mathrm{mm}}^{2}$) follows a power‐law, decaying as $1/\sqrt{b}$. It has also been shown, theoretically, that for planar tensor encoding (PTE), the direction‐averaged diffusion‐weighted MRI signal decays as 1/*b*. We aimed to confirm this theoretical prediction in vivo. We then considered the direction‐averaged signal for arbitrary b‐tensor shapes and different tissue substrates to look for other conditions under which a power‐law exists.

**Methods:**

We considered the signal decay for high b‐values for encoding geometries ranging from 2‐dimensional PTE, through isotropic or spherical tensor encoding to LTE. When a power‐law behavior was suggested, this was tested using in silico simulations and, when appropriate, in vivo using ultra‐strong (300 mT/m) gradients.

**Results:**

Our in vivo results confirmed the predicted 1/*b* power law for PTE. Moreover, our analysis showed that using an axisymmetric b‐tensor a power‐law only exists under very specific conditions: (a) “stick‐like” tissue geometry and purely LTE or purely PTE waveforms; and (b) "pancake‐like" tissue geometry and a purely LTE waveform.

**Conclusions:**

A complete analysis of the power‐law dependencies of the diffusion‐weighted signal at high b‐values has been performed. Only three specific forms of encoding result in a power‐law dependency, pure linear and pure PTE when the tissue geometry is “stick‐like” and pure LTE when the tissue geometry is "pancake‐like". The different exponents of these encodings could be used to provide independent validation of the presence of different tissue geometries in vivo.

## 1 INTRODUCTION

Diffusion MRI (dMRI) provides a tool to study brain tissue based on the Brownian motion of water molecules^1^ and is sensitive to differences in the microstructure of the tissue.^2^, ^3^, ^4^ Different mathematical representations have been proposed to describe the relationship between the diffusion signal, the strength of diffusion‐weighting (b‐value), and the microstructural properties of the tissue under investigation.^5^, ^6^, ^7^ The most prominent are the biexponential,^8^, ^9^, ^10^, ^11^, ^12^ the stretched exponential,^13^ and the power‐law.^14^, ^15^, ^16^, ^17^ The mathematical forms of these approaches are quite different. In the biexponential approach, the large b‐value behavior is assumed to be dominated by the intracellular compartment. For stretched exponentials, the signal relationship with the b‐value is $\exp\left[-{\left(kb\right)}^{a}\right]$, where *k* is a constant and *a* < 1 is the stretching parameter. In the statistical model developed by Yablonskiy et al,^14^ the signal decays as 1/*b* for large *b*, while the other studies^15^, ^16^, ^17^ have reported that the signal at high b‐values decays as $1/\sqrt{b}$.

The aforementioned studies all used the conventional (Stejskal‐Tanner) pulsed‐gradient diffusion encoding,^18^ where diffusion sensitization occurs along a single axis. Since the development of the pulsed gradient spin echo sequence,^18^ there have been many works aimed at maximizing the information that can be obtained from a dMRI experiment by exploring different acquisition protocols.^19^, ^20^ One such modification is the addition of multiple gradient pairs. We can use two pairs of pulsed‐field gradients to obtain a double diffusion encoding.^21^, ^22^ It has been shown that double diffusion encoding, as well as other multiple encoding schemes such as triple diffusion encoding,^23^ provide information that is not accessible with single diffusion encoding.^24^

This approach has been utilized by several groups for extracting microstructural information.^25^, ^26^, ^27^, ^28^, ^29^ A framework was recently proposed^30^ to probe tissue using different q‐space trajectory encodings which can be described by a second‐order b‐tensor. Single, double and triple diffusion encoding can be characterized by b‐tensors, with one, two, and three non‐zero eigenvalues, respectively. In this framework, single diffusion encoding is also called linear tensor encoding (LTE), double diffusion encoding with perpendicular directions is called planar tensor encoding (PTE) and triple diffusion encoding with three equal eigenvalues is called spherical tensor encoding (STE).

In this study, we investigate the effect of different b‐tensor encodings on the diffusion signal at high b‐values. To remove the effect of fiber orientation distribution,^31^ the acquired signal is averaged over all diffusion directions for each shell. This so‐called powder‐averaged signal^32^, ^33^ has less complexity than the direction‐dependent signal. Powder averaging yields a signal whose orientation‐invariant aspects of diffusion are preserved but with an orientation distribution that mimics complete dispersion of anisotropic structures.

In this work, we confirm in vivo the theoretical prediction^34^ for PTE that the direction‐averaged signal decays as 1/*b*. We then consider, more generally, the direction‐averaged signal for arbitrary b‐tensor shapes and different tissue substrates to determine the conditions under which the power‐law exists. We establish the range of b‐values over which we observe any power‐law scaling, including considerations of signal amplitude compared to the noise, and the impact of the number of encoding directions on any power‐law decay. Finally, we consider how observation of a power‐law signal dependence with more than one gradient wave‐form could help to provide a “cross‐validation” for specific tissue geometries.

## 2 THEORY

In multi‐dimensional diffusion MRI, the b‐matrix is defined as an axisymmetric second‐order tensor, $B\;=\;b/3\left(1-b_{\Delta }\right)I_{3}\;+\;bb_{\Delta }gg^{T}$, where **g** is the diffusion gradient direction and the b‐value, *b*, is defined as the trace of the b‐matrix. The eigenvalues of the b‐matrix are $b_{\|}$, $b_{⊥}^{\left(1\right)}$ and $b_{⊥}^{\left(2\right)}$ where $b_{⊥}^{\left(1\right)}\;=\;b_{⊥}^{\left(2\right)}\;=\;b_{⊥}$ and $b_{\|}$ is the largest. $b_{\Delta }$ is defined as $b_{\Delta }\;=\;\left(b_{\left|\right|}-b_{⊥}\right)/\left(b_{\left|\right|}\;+\;2b_{⊥}\right)$. Changing $b_{\Delta }$, we can generate different types of b‐tensor encoding. For LTE, PTE, and STE, $b_{\Delta }\;=\;1,\;-1/2$, and 0, respectively.^23^

For the powder‐averaged signal, the diffusion attenuation is a function of the orientation‐invariant aspects of the diffusion and the encoding. The compartment diffusion attenuation is (Equation (34) in^35^):

(1) $$S\left(b\right)\;=\;\frac{\sqrt{\pi }e^{-\frac{b}{3}\left(D^{\|}+2D^{⊥}-b_{\Delta }\left(D^{\|}-D^{⊥}\right)\right)}\;\text{erf}\left(\sqrt{bb_{\Delta }\left(D^{\|}-D^{⊥}\right)}\right)}{2\sqrt{bb_{\Delta }\left(D^{\|}-D^{⊥}\right)}}$$

where *S* is the normalized diffusion signal and $D^{\|}$ and $D^{⊥}$ are the parallel and perpendicular diffusivities, respectively. We use the subscript “ ${}_{e}$” and “ ${}_{a}$” to denote parameters of the extra‐axonal and the axonal compartments, respectively.

Here, we study the effect of axisymmetric b‐tensor shape on the diffusion‐weighted signal at high b‐values.

### 2.1 Linear, planar and STE

In LTE, $b_{\Delta }\;=\;1$ and assuming stick‐like geometry, $D^{⊥}\;=\;0$ in Equation (1), therefore $S_{\mathrm{ic}}\;\propto \;b^{-1/2}$. The sensitivity of MR to axon radius would alter the $b^{-1/2}$ scaling^36^ because there will be a perpendicular diffusivity and the exponent in Equation (1) would not equal zero, and thus the exponential term outside of the error function would not equal unity.

In PTE, $b_{\Delta }\;=\;-1/2$ and $S_{\mathrm{ic}}$ has the following form:

(2) $$S_{\mathrm{ic}}^{\mathrm{PTE}}\left(b\right)\;=\;\frac{\sqrt{\pi }e^{\frac{-b{D_{a}}^{\left|\right|}}{2}}\;\text{erfi}\left(\sqrt{b{D_{a}}^{\left|\right|}/2}\right)}{2\sqrt{b{D_{a}}^{\left|\right|}/2}}$$

**Figure 1:**
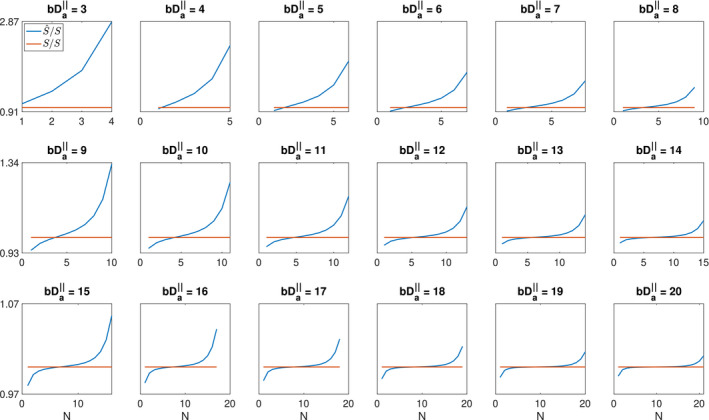
The approximated signal over the original PTE signal ( $\hat{S}/S$), for different *N* values

For large b‐values, $b{D_{a}}^{\left|\right|}\;\gg \;1$; therefore, the diffusion signal can be approximated by the following equation (see Appendix A):

(3) $$S_{\mathrm{ic}}^{\mathrm{PTE}}\left(b\right)\approx \frac{1}{b{D_{a}}^{\left|\right|}}\sum_{k\;=\;0}^{N}\frac{(2k-1)!!}{{\left(b{D_{a}}^{\left|\right|}\right)}^{k}}$$

where !! denotes the double factorial and *N* depends on the $b{D_{a}}^{\left|\right|}$ value (Figure 1 and Table 1).

**Table 1:** The minimum number of terms for reconstructing the PTE signal for different error threshold values

	$b{D_{a}}^{\|}$																		
		3	4	5	6	7	8	9	10	11	12	13	14	15	16	17	18	19	20
Error threshold	0.06	1	1	1	1	1	1	1	1	1	1	1	1	1	1	1	1	1	1
	0.05	–	1	1	2	2	1	1	1	1	1	1	1	1	1	1	1	1	1
	0.04	–	1	2	2	2	2	2	1	1	1	1	1	1	1	1	1	1	1
	0.03	–	1	2	2	2	2	2	2	1	1	1	1	1	1	1	1	1	1
	0.02	–	1	–	2	2	2	2	2	2	2	2	1	1	1	1	1	1	1
	0.01	–	–	–	2	3	3	3	3	3	3	2	2	2	2	2	1	1	1

For large b‐values, the extra‐axonal signal decays exponentially faster than the intra‐axonal compartment, $\exp\left(-b{D_{e}}^{⊥}\right)\ll 1$, and can be neglected.

The asymptotic expansion of erfi(*x*) in Equation A2 (see Appendix A) is valid when *x* → ∞, but large values of $b{D_{a}}^{\|}$ would suppress the signal to immeasurable levels, and therefore there are practical bounds on the value of $b{D_{a}}^{\|}$ that can be achieved. Therefore, we compared the original signal in Equation 2 and the approximated signal using Equation 3 for different values of *N* and $b{D_{a}}^{\|}$ (Figure 1 and Table 1). We use a normalized error to compare the original (Equation 2) and the approximated signal (Equation 3):

(4) $$\text{Normalized error}\;=\;\frac{|S-\hat{S}|}{S}\;=\;\left|1-\frac{\hat{S}}{S}\right|$$

where *S* is the original signal obtained from Equation 2 and $\hat{S}$ is the approximated signal from Equation 3.

In STE, $b_{\Delta }\;=\;0$ and $S_{\mathrm{ic}}\;=\;\exp\left(-\frac{b}{3}{D_{a}}^{\left|\right|}\right)$. For large b‐values, both intra‐ and extra‐axonal signals decay exponentially fast, $\exp\left(-\frac{b{D_{a}}^{\|}}{3}\right)\ll 1$, $\exp\left(-\frac{b({D_{e}}^{\|}+2{D_{e}}^{⊥})}{3}\right)\ll 1$ and both of them are negligible. Therefore, the STE does not provide a considerable signal for large b‐values in a two‐compartment model.

### 2.2 General case of axisymmetric B‐tensor

Here, we consider the general case of an axisymmetric b‐tensor $b_{\Delta }\;\neq \;0$ to cover all b‐tensor shapes between $b_{\Delta }\;=\;-0.5$ (PTE) to $b_{\Delta }\;=\;1$ (LTE).

#### 2.2.1 $0\;<\;b_{\Delta }\;\leq \;1$

As noted above, in this range, the error function in Equation (1) goes to 1 for high b when $D^{\|}\;\neq \;0$. In this case, to have a power‐law relationship between the signal and the b‐value, the exponential term $\exp[-b\left(D^{\|}+2D^{⊥}-b_{\Delta }\left(D^{\|}-D^{⊥}\right)\right)/3]$ should go to one and therefore $D^{\|}+2D^{⊥}-b_{\Delta }\left(D^{\|}-D^{⊥}\right)\;=\;0$. For $D^{\|}\;\neq \;0$, $D^{⊥}/D^{\|}\;=\;\left(b_{\Delta }-1\right)/\left(b_{\Delta }+2\right)$ which is only physically plausible (i.e., the ratio of diffusion coefficients has to be ≥0) for $b_{\Delta }-1\;\geq \;0$, but the maximum value that $b_{\Delta }$ can take is one, and therefore $D^{⊥}$ has to be zero, that is, the tissue geometry has to be that of a stick, and the b‐tensor has to be a pure LTE to have a power‐law relationship. If $D^{\|}\;=\;0$ then we have the imaginary error function and therefore there is a power‐law relationship for the pancake‐like tissue geometry.

#### 2.2.2 $-0.5\;\leq \;b_{\Delta }\;<\;0$

Conversely, in the range $-0.5\;\leq \;b_{\Delta }\;<\;0$, as in Equation (2), the error function becomes imaginary. Similar to the first scenario, to have a power‐law relationship the exponential term has to be one. By replacing the first term of the approximation in Equation (A2) into Equation (1), we have:

(5) $$S\left(k\;=\;0\right)\;\approx \;\frac{e^{\frac{-b}{3}\left[\left(D^{\|}+2D^{⊥}\right)-b_{\Delta }\left(D^{\|}-D^{⊥}\right)\right]-bb_{\Delta }\left(D^{\|}-D^{⊥}\right)}}{-2bb_{\Delta }\left(D^{\|}-D^{⊥}\right)}$$

To have the exponential equal to one:

(6) $$\frac{D^{⊥}}{D^{\|}}\approx \frac{2b_{\Delta }+1}{2b_{\Delta }-2}$$

where the right side of the equation is negative for $-0.5\;<\;b_{\Delta }\;<\;0$ which is not physically plausible for the left side of the equation (i.e., ratio of diffusivities). Therefore, the only possible case is to have $D^{⊥}\;=\;0$ which again means stick‐like tissue geometry and $b_{\Delta }\;=\;-0.5$ which is pure PTE. Clearly the exponential term will become zero if and only if $b_{\Delta }\;=\;-0.5$, and thus, the 1/*b* signal form will occur if and only if the b‐tensor shape has just 2 non‐zero eigenvalues, that is, pure PTE. Thus, for stick‐like geometries, there are only two b‐tensor shapes for which a power‐law exists: pure linear and pure planar. Moreover, for pancake‐shape tissue geometries, a power‐law exists if and only if the encoding geometry is pure LTE.

Herberthson et al^34^ have also considered the signal for arbitrary waveforms, and provided a theoretical prediction of a 1/*b* power law. The $S\;\propto \;b^{-1}$ dependence is valid for an intermediate range of diffusion weightings while the asymptotic behavior of the signal decay is determined by a steeper decay.^37^

## 3 METHOD

### 3.1 Simulations

Synthetic data were generated with 60 diffusion encoding gradient orientations uniformly distributed on the unit sphere^38^, ^39^ and 21 b‐values spaced in the interval [0, 10 000 $s/{\mathrm{mm}}^{2}$] with a step‐size of 500 $s/{\mathrm{mm}}^{2}$. The noise is considered Rician with SNR = 150 for the b0 image, which is practically feasible using the Connectom scanner with an echo time of 88 ms.^40^ A three‐compartment model with a Watson orientation distribution function is used:

(7) $$S/S_{0}\;=\;f_{1}\int_{S^{2}}W\left(n\right)S_{\mathrm{cyl}}\left(n\right)dn\;+\;f_{2}\int_{S^{2}}W\left(n\right)S_{\mathrm{ec}}\left(n\right)dn\;+\;f_{3}S_{\mathrm{sph}}\left(R_{s}\right)$$

where $f_{1}$, $f_{2}$, and $f_{3}$ are the intra‐axonal, extra‐axonal, and the sphere signal fraction respectively, *W*(**n**) is the Watson ODF, $S_{\mathrm{ec}}$ is the extra‐axonal signal, $S_{\mathrm{cyl}}$ is the signal attenuation of the impermeable cylinders^41^ and $S_{\mathrm{sph}}$ is the restricted diffusion inside the spherical compartment in the presence of b‐tensor encoding^42^ (Appendix B). The ground truth parameter values defined by a set of parameters [ $f_{1}$ = 0.65, ${D_{a}}^{\|}\;=\;2\;\mu m^{2}/\text{ms}$, $D_{e}^{\|}\;=\;2\;\mu m^{2}/\mathrm{ms}$, $D_{e}^{⊥}\;=\;0.25,\;0.5,\;0.75\;\mu m^{2}/\text{ms}$ and *κ* = 11] and axon radius $r_{i}$, come from the bins of the histograms in.^43^ We average the signal over all $r_{i}$s weighted by $r_{i}^{2}$. In histology, there is a possibility of tissue shrinkage. To account for this change, the axon radius values are multiplied with three shrinkage factors *η* = 0, 1, 1.5.^43^, ^44^ The *η* = 0 case simulates the effect of zero‐radius axons.

The third compartment is simulated as a sphere with zero radius (dot) and a sphere with radius $R_{s}\;=\;8\;\mu m$ to consider the effect of combining the environments on the power‐law scaling.

The noisy diffusion‐weighted signal is modeled according to the following:

(8) $$S_{n}\;=\;\sqrt{{\left(S+N_{r}\left(0,\;\sigma \right)\right)}^{2}+N_{i}{\left(0,\;\sigma \right)}^{2}}$$

where $S_{n}$ and *S* are the noisy and noise‐free signal, respectively, and $N_{r}$ and $N_{i}$ are the normal distributed noise in the real and imaginary images respectively with a standard deviation of *σ*.^45^, ^46^ The Matlab code for the simulation is available on GitHub (https://github.com/maryamafzali/PTE_Cylinder-).

### 3.2 In vivo data

Two healthy participants who showed no evidence of a clinical neurologic condition were scanned in this study that was conducted with approval of the Cardiff University School of Psychology ethics committee. Diffusion‐weighted images were acquired with 60 gradient directions for PTE on a 3T Connectom MR imaging system (Siemens Healthineers, Erlangen, Germany). Twenty axial slices with a voxel size of 4 mm isotropic (given the strong signal attenuations investigated here, a low resolution of 4 mm isotropic was used) and a 64 × 64 matrix size, TE = 88 ms, TR = 3000 ms, were obtained for each individual.

**Figure 2:**
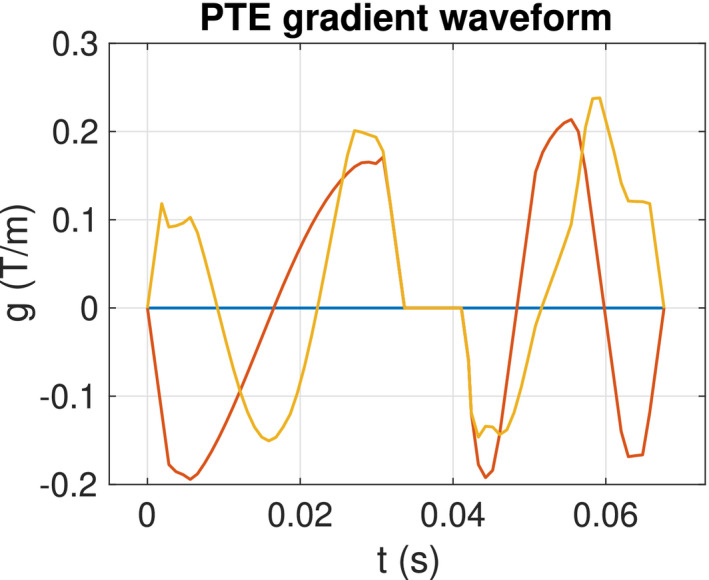
The gradient waveform of the planar tensor encoding

To take full advantage of q‐space trajectory imaging, it is imperative to respect the constraints imposed by the hardware, while at the same time maximizing the diffusion encoding strength. Sjolund et al^47^ provided a tool for achieving this by solving a constrained optimization problem that accommodates constraints on maximum gradient amplitude, slew rate, coil heating, and positioning of radio frequency pulses. The gradient waveform is obtained based on a framework that maximizes the b‐value for a given measurement tensor and echo time (Figure 2). Substantial gains in terms of reduced echo times and better signal‐to‐noise ratio can be achieved, in particular as compared with naive PTE.

Diffusion data were acquired for 10 b‐value shells from 1000 to 10 000 $s/{\mathrm{mm}}^{2}$ with a step size of 1000 $s/{\mathrm{mm}}^{2}$ and each shell had the same 60 diffusion encoding gradient orientations uniformly distributed on the unit sphere. One b0 image was acquired between each b‐value shell as a reference.

The data were denoised^48^ and corrected for Gibbs ringing,^49^ eddy current distortions, subject motion,^50^ and gradient nonlinearity.^51^ We normalized the direction‐averaged signal based on the b0 signal in each voxel.

In order to find the minimum number of directions necessary to have a rotationally invariant signal powder average, we used the method proposed by Szczepankiewicz et al.^52^ The diffusion was assumed Gaussian, and was described by an axially symmetric diffusion tensor, defined by $D\;=\;rr^{T}\left(D^{\|}-D^{⊥}\right)+D^{⊥}I$, where $D^{\left|\right|}$ and $D^{⊥}$ are the parallel and perpendicular diffusivity, *r* is the tensor principal direction and *I* is the identity matrix. We used *S* =  exp (−*trace*(*BD*)) to simulate diffusion‐weighted signal for linear and PTE. The b‐matrix for LTE and PTE is defined as $B_{\mathrm{LTE}}\;=\;bgg^{T}$, and $B_{\mathrm{PTE}}\;=\;b/2\left(I-gg^{T}\right)$, respectively, where g is the gradient direction and the b‐value, *b*, is the trace of b‐matrix. The orientation, *r*, was rotated in 512 different directions to consider the effect of rotation. We used $b\;=\;7000-10\;000\;s/{\mathrm{mm}}^{2}$ with a step size of $1000\;s/{\mathrm{mm}}^{2}$. We change $D^{\left|\right|}$ and $D^{⊥}$, while keeping the mean diffusivity of $MD\;=\;1.0\;\mu m^{2}/\mathrm{ms}$ and set FA = 0.95 because the impact of rotational variance is most pronounced at high anisotropy. The direction averaged signal was calculated and the coefficient of variation (CV) across all 512 orientations was estimated using *CV* = *SD*/*E* where *SD* and *E* are the standard deviation and mean value, respectively. The threshold was set to *CV* < 0.01 and the minimum number of directions to meet this condition was calculated.

## 4 RESULTS

Figure 1 shows $\hat{S}/S$ for $3\;<\;b{D_{a}}^{\|}\;<\;20$ and 4 < *N* < 21. The selected range of $b{D_{a}}^{\|}$ is compatible with the range of b‐values that we can obtain from the Connectom scanner and also the range of ${D_{a}}^{\|}$ that exists in the brain.^53^ Based on Figure 1 the number of terms in Equation 7 should be smaller than or equal to the $b{D_{a}}^{\|}$ ( $N\leq \lfloor b{D_{a}}^{\|}\rfloor$ where ⌊...⌋ denotes the floor function) to have the minimum error ( $\hat{S}/S$ is close to one). As the number of terms goes beyond the $b{D_{a}}^{\|}$, the error increases. Table 1 shows the minimum number of terms, *N*, for different error threshold values (0.01‐0.06). When the error threshold is 0.02, we can approximate Equation 2 with the first term in Equation 3 if $b{D_{a}}^{\|}\geq 14$. For the error threshold of 0.06, the maximum $b{D_{a}}^{\|}$ to approximate the signal with the first term is 3.

Diffusion MRI is an inherently low SNR measurement technique, particularly when strong diffusion weightings are utilized. To reach the level that enables us to approximate the planar diffusion signal in Equation 2 with the first term of Equation 3, we need to use relatively high b‐values ( $b{D_{a}}^{\|}\geq 14$). One of the challenges with the high b‐values is the noise, as the signal amplitude can be close to the noise floor. Therefore, here we find the maximum value of $b{D_{a}}^{\|}$ that we can use before hitting this rectified noise floor (see Appendix C).

The noise in complex MR data is normally distributed, whereas the noise in magnitude images is Rician distributed.^45^, ^46^ Here, we select a minimum SNR value equal to 2 (see Appendix C). By setting the diffusion‐weighted intensity to the mean background signal, we obtain the b‐value that makes the signal equal to the noise floor.

**Figure 3:**
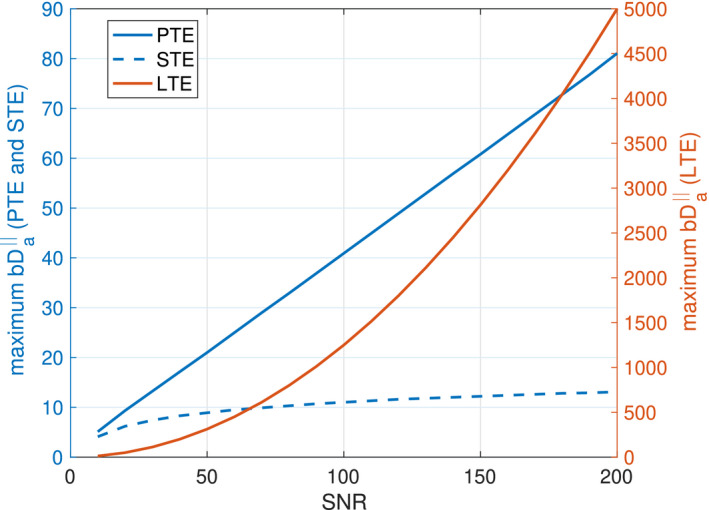
Maximum $b{D_{a}}^{\|}$ vs SNR. The Maximum $b{D_{a}}^{\|}$ value is proportional to the square of SNR, ( $b{D_{a}}^{\|}∼SNR^{2}$) for LTE, where this relationship is linear for PTE ( $b{D_{a}}^{\|}∼SNR$) and it is logarithmic for STE ( $b{D_{a}}^{\|}∼\ln\left(SNR\right)$)

Figure 3 shows the maximum $b{D_{a}}^{\|}$ as a function of SNR for different encoding schemes and different noise floors. The maximum value of $b{D_{a}}^{\|}$ that can be used while staying above the noise floor increases when SNR increases, but the rate of this change is different for different encoding schemes. The maximum $b{D_{a}}^{\|}$ value ( ${b{D_{a}}^{\|}}^{\max}$) is proportional to the square of SNR, ( ${b{D_{a}}^{\|}}^{\max}∼SNR^{2}$) for LTE, where this relationship is linear for PTE ( $b{D_{a}}^{\|}∼SNR$) and it is logarithmic for STE ( $b{D_{a}}^{\|}∼\ln\left(SNR\right)$). Based on this plot, if SNR = 50 the values of ${b{D_{a}}^{\|}}^{\max}$ for linear, planar and STE schemes are around 312, 21 and 9 respectively. The SNR in our data is around 150 therefore the measured signal values in our experiment are higher than the noise level. For this SNR, the ${b{D_{a}}^{\|}}^{\max}$ for linear, planar and STE schemes are around 15 625, 100 and 16 respectively.

**Figure 4:**
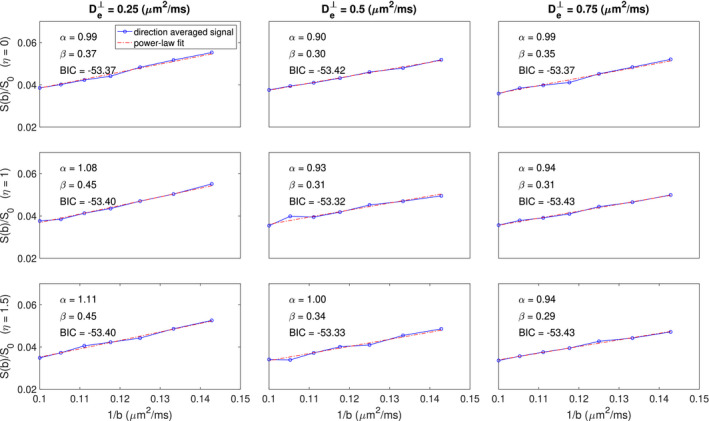
Simulated direction‐averaged PTE signal for $7000\;<\;b\;<\;10\;000\;s/{\mathrm{mm}}^{2}$ and the results of the power‐law fit

Figure 4 shows the simulated direction‐averaged PTE signal ( $f_{3}\;=\;0$) as a function of 1/*b* for three different perpendicular diffusivities and three different shrinkage factors. The result of the power‐law fit ( $S\;=\;\beta b^{-\alpha }$) is represented by the red dashed line and the *α* and *β* values are reported in each plot. The trust‐region‐reflective algorithm is used for optimization with a fixed initial value (*α* = 1 and *β* = 0.2). The goodness of fit is evaluated using the Bayesian information criterion (BIC).^54^ In our simulation, *f* = 0.65, ${D_{a}}^{\|}\;=\;2\;\mu m^{2}/\mathrm{ms}$, therefore if the approximation in Equation 3 is valid, *β* ≈ 0.325 and *α* ≈ 1 indicate that the fit approximately matches the theory.

**Figure 5:**
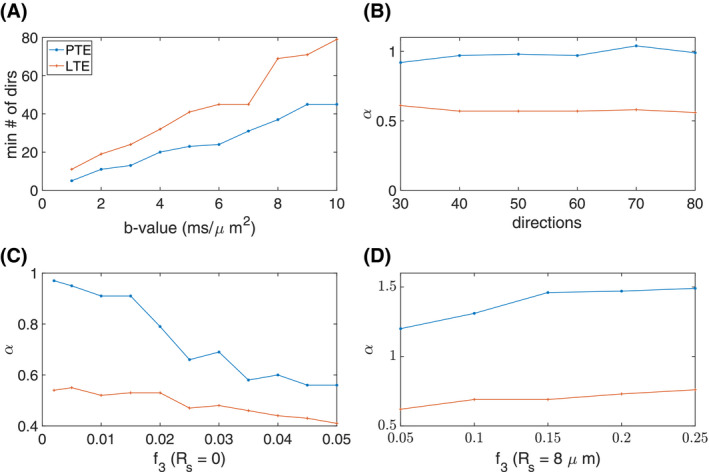
A, The minimum number of directions for a rotationally invariant powder average signal at different b‐values, B, the changes of power‐law scaling versus number of gradient directions, C, the changes of the power‐law scaling (*α*) versus “still water” signal fraction, and D, the changes of the power‐law scaling (*α*) versus sphere signal fraction for PTE compared to LTE

Szczepankiewicz et al^52^ showed that PTE needs a lower number of directions (15‐20 directions for $b\;<\;\;=\;4000\;s/{\mathrm{mm}}^{2}$) compared to LTE (20‐32 directions $b\;<\;\;=\;4000\;s/{\mathrm{mm}}^{2}$), to provide a rotationally invariant signal powder average, making it more efficient for achieving rotational invariance. Figure 5A shows the minimal number of encoding directions required to obtain rotational invariance over a broader range of b‐values ( $1000\;<\;b\;<\;10\;000\;s/{\mathrm{mm}}^{2}$). This was obtained using the method proposed by Szczepankiewicz et al.^52^ The plots for LTE and PTE are divergent, meaning that the relative efficiency of PTE over LTE increases with the b‐value used. Indeed, for the range of b‐values used in this work, ( $7000\;<\;b\;<\;10\;000\;s/{\mathrm{mm}}^{2}$), the minimum number of encoding directions for rotational invariance is 45 for PTE and almost 80 for LTE.

Figure 5B illustrates the changes of exponent *α* using LTE compared to PTE. An insufficient number of diffusion directions in powder averaging may cause the break of power‐law scaling. Therefore, we have to consider this when computing a powder average at very high b‐values. In Figure 5B, even with a large number of directions the *α*‐values deviate considerably from the theoretical value (especially for LTE). The deviation is most likely attributable to the non‐zero perpendicular diffusivity, and also the shrinkage factor, *η*, chosen for the simulations. In Figure 5, we used *η* = 1.5 and $D_{e}^{⊥}\;=\;0.75\;\mu m^{2}/\mathrm{ms}$ matching the results of simulations in.^17^ The still water, or “dot” compartment has a diffusivity close to zero, which can affect the power‐law scaling. Figure 5C shows the changes of the power‐law scaling, *α*, versus “still water” (a.k.a. “dot”) signal fraction for PTE and LTE on the simulated data with $D^{⊥}\;=\;0.75\;\left(\mu m^{2}/\mathrm{ms}\right)$. Note that over the $f_{3}$ range of $0\;<\;f_{3}\;<\;0.02$, the *α* for LTE doesn’t change by more than two percent, and thus is relatively insensitive to the presence of the still water fraction. In contrast, the PTE profile shows a much stronger dependence on the dot water fraction, with a rapid deviation from the “pure stick”, *α*‐value of 1, as the dot fraction increases.

**Figure 6:**
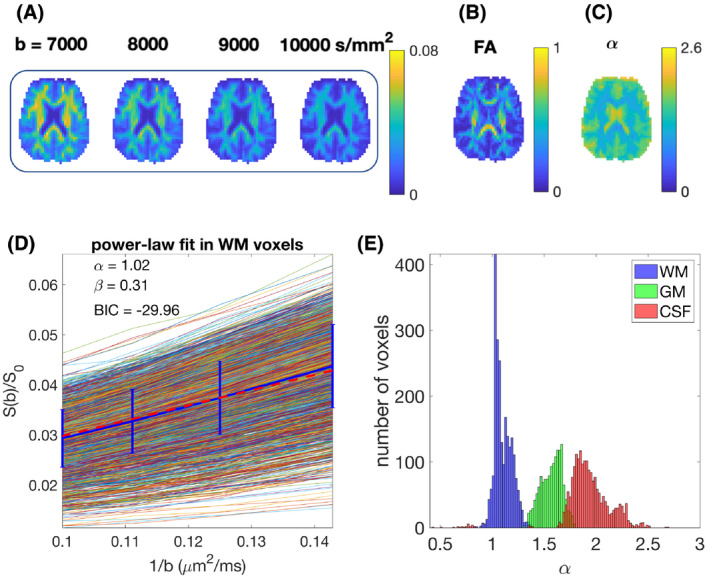
A, Direction‐averaged diffusion signal for different b‐values (*b* = 7000 to 10 000 $s/{\mathrm{mm}}^{2}$) in PTE, B, FA, C, parametric map of the exponent *α*. D, The plot of the diffusion signal vs 1/*b* for in vivo white matter voxels using planar tensor encoding. The blue curve with the error bar shows the mean and the std of the average signal and the red line shows the power‐law fit. The parameters, *α* and *β* are reported in the figure. *α* = 1 shows the power‐law relationship between the diffusion signal and the b‐value. E, The histogram of *α* values

Figure 5D shows the changes in the exponent *α* in the presence of a spherical compartment (i.e., diffusion restricted inside a spherical space) with a radius of $R_{s}\;=\;8\;\mu m$. The estimated values of alpha from these simulations are in the range of 1.2‐1.5, increasing monotonically with $f_{3}$. This range is remarkably close to the values found in vivo in gray matter, which were in the range 1.2‐1.7 (see Figure 6), confirming that signal decay in the gray matter can be represented by a combination of stick‐like and spherical compartments.^55^

A rough estimation of the SNR was performed, using pixels from the signal area and from the background, $SNR\;=\;\left(\left\langle M_{S}\left(x\right)\right\rangle \right)/\left(\sqrt{2/\pi }\left\langle M_{B}\left(x\right)\right\rangle \right)$ where 〈.〉 denotes the average operator. The estimation of the signal was performed using the average of several ROIs (with 4 voxels) with a high direction‐averaged signal, $M_{S}\left(x\right)$, for the same b‐value. (Note that, although the average is a biased estimator of the signal for Rician data, for the case of higher SNR areas, this bias must be small). The parameter of noise *σ* is estimated from only noise pixels in the background, where the signal is known to follow a Rayleigh distribution. Using the mean of a Rayleigh, $E\left\{M\left(x\right)\right\}\;=\;\sqrt{\pi /2}\sigma$, we can estimate $\hat{\sigma }\;=\;\sqrt{2/\pi }\left\langle M_{B}\left(x\right)\right\rangle$. SNR in WM ranged from 7.5 to 10.3 for $b\;=\;10\;000\;s/{\mathrm{mm}}^{2}$ and between 130 and 150 for the non‐diffusion weighted image (*b* = 0).

Figure 6 illustrates the normalized direction‐averaged diffusion signal of the in vivo data for different b‐values (7000 ≤ *b* ≤ 10 000), the FA and *α* map, the power‐law fit over white matter voxels, and the histogram of the *α* values in white matter, gray matter, CSF. For high b‐values ( $7000\;<\;b\;<\;10\;000\;s/{\mathrm{mm}}^{2}$), the amount of the signal is considerable compared to the noise and the white matter structure is completely clear in the images because of high SNR.

The results show that the data are well described by power‐law behavior, with *α* ≈ 1 which confirms the validity of the signal approximation using the first term in Equation 3.

The constant term *β* is estimated from the fitting of equation $S/S_{0}\;=\;\beta b^{-\alpha }$ and based on Equation (3) $\beta \approx f/D_{a}^{\left|\right|}$. In Figure 6, we have *β* = 0.31. If we assume the intracellular signal fraction, *f*, is around 0.6 then the intra‐neurite diffusivity $D_{a}^{\left|\right|}$ will be around $2\;\mu m^{2}/\mathrm{ms}$, which is feasible. To segment the brain image into different tissues, we used FAST (FMRIB’s Automated Segmentation Tool) in FSL.^56^ In the WM, the *α* value is close to one, supporting the theory. In gray matter and CSF, the exponent is larger (1.5 and 2, respectively). According to the theory outlined above, this would be consistent with a lack of pure “stick‐like” geometry in these tissue components. The spatial resolution of the data must be recognized, that is, with 4 mm isotropic voxels, obtaining a “pure” GM signal and “pure” CSF signal is challenging. It is likely that the intermediate exponent in the GM between that of the WM and CSF is partly attributable to a partial volume effect, and partly attributable to the inadequacy of the model for gray matter architecture. The *α*‐value in the gray matter seems surprising because a substantial portion of gray matter is composed of neurites where the stick model seems plausible. The deviation away from an *α*‐value of 1 could, however, potentially be explained by differences in water exchange times between white and gray matter. For example, if the exchange time in gray matter is comparable to the diffusion time of our experiment, perhaps as a result of high permeability (compared to in white matter), then the “stick‐like” behavior would not be observed, leading to a deviation of the *α*‐value away from unity. The exponent in gray matter is similar to the one obtained using the combination of “stick + sphere”.^55^ Further investigation of this phenomenon in gray matter is beyond the scope of this work.

Table 2 shows the mean and the standard deviation of exponent *α* in white matter, gray matter and CSF for two different subjects. The mean value in WM is around one, in the gray matter it is around 1.5 and for CSF around 2.

**Table 2:** The mean and the standard deviation of the exponent *α* in white matter, gray matter and CSF

	WM	GM	CSF	BIC
Subject 1	1.1054 ± 0.085	1.5716 ± 0.1009	1.9004 ± 0.2968	−29.9676
Subject 2	1.1100 ± 0.1447	1.6617 ± 0.1836	2.0469 ± 0.2250	−30.293

## 5 DISCUSSION

The main findings of this paper are twofold: (1) confirmation in silico and in vivo, of a power‐law relationship between the direction‐averaged DWI signal and the b‐value using PTE, as given by Equation 3, for b‐values ranging from 7000 to 10 000 $s/{\mathrm{mm}}^{2}$. In white matter, the average value of the estimated exponent is around one; and (2) proof that there are only 3 specific conditions under which a power‐law exists (for axisymmetric b‐tensor): pure LTE or PTE for pure stick‐like geometry and pure LTE for pure pancake‐like geometry. To the best of our knowledge, no pure pancake‐like geometries exist in the human brain and so in vivo validation of this latter result is impossible.

Returning to the case of a power‐law with PTE, for smaller b‐values, this behavior must break down as the DWI signal of PTE cannot be approximated by Equation 3 (Figure 1) and also we cannot neglect the contribution of the extracellular compartment. It could also fail for very large b‐values, if there were immobile protons that contributed a constant offset to the overall signal or if there is any sensitivity to the axon diameter.^36^ Besides, if we do not have a sufficient number of diffusion directions for powder averaging, this power‐law scaling can break.

The exponent of approximately one for white matter using PTE is consistent with the large b‐value limit (in the range of b‐values, used here) predicted for a model of water confined to sticks (Equation 3), which is used to describe the diffusion dynamics of intra‐axonal water. Our results confirm this relationship between the diffusion signal and the b‐value (Figure 6 and Table 2).

The $b^{-1/2}$‐scaling for LTE has previously been suggested by^16^, ^17^. We should emphasize that the power‐law behavior with the exponent of 1/2 was only found in white matter in LTE. Two other proposed models predict power law signal decay, for large b‐values using a LTE. One of these is the statistical model,^14^ where the signal decays as 1/*b* for large *b*. Some other models,^57^, ^58^, ^59^ assume a gamma distribution for the diffusion coefficients and a family of Wishart distributions.^60^ However, in this case, the exponent does not have a universal value, it depends on the distribution.

This work interprets the diffusion‐weighted MRI signal decay at high b‐values in the form of $S∼b^{-1}$ for PTE, this power‐law relationship is also reported by Herberthson et al.^34^ An important application of this finding is using the combination of linear and PTEs to characterize the intra‐axonal diffusivity and the signal fraction as it is proposed by^61^ using triple diffusion encoding. As discussed earlier, the results in Figure 5C show that the *α* value for PTE is much more sensitive to the inclusion of a still‐water compartment than LTE. If using LTE alone, one might erroneously conclude from an alpha value very close to 0.5, that only stick‐like geometries exist—even in the presence of a non‐negligible dot fraction. In contrast, as seen in Figure 5C *α* is only close to 1 when the still‐water component is zero, which confers an advantage of PTE over LTE for identifying pure stick‐like geometry. This likely arises because at a given high b‐value the proportional contribution to the total signal from the dot‐compartment is larger for the PTE signal (decays as 1/*b*) than it is for the LTE signal (decays as $1/\sqrt{b}$). One might therefore consider using PTE alone for identifying pure stick‐like geometry. However, given that the SNR per unit b is, by definition, much lower with PTE than with LTE, we recommend that PTE and LTE are used in combination as a way of “cross‐validating” pure stick‐like geometry. Finally, we observed a larger alpha value in the gray matter than in white matter. This difference in exponents was previously reported for LTE by McKinnon et al,^16^ who also reported higher exponent in gray matter than in white matter. In our work, using PTE, the higher alpha value could be explained by modeling the signal as arising from both stick‐like and spherical compartments. It is possible that other models might explain this signal decay more accurately, but this is beyond the scope of the current work.

## 6 CONCLUSION

This work explores the diffusion‐weighted MRI signal decay at high b‐values for PTE and STE complementing and extending previous works on LTE. By exploring diffusion averaged signals, we conclude that the signal from STE decays exponentially for all the range of b‐values. The intra‐axonal signal does not decay exponentially as a function of b for linear and planar tensor encoding in high b‐values. The direction‐averaged DWI signal of PTE and LTE decreases with increasing b‐values as a power law, for b‐values ranging from 7000 to 10 000 $s/{\mathrm{mm}}^{2}$. In white matter, the in vivo exponent characterizing this decrease is close to one‐half, for LTE and one for PTE. These experimental results are consistent with theoretical predictions for the signal decay at large b‐values for tissue in which axonal water diffusion is confined to sticks when there is no sensitivity to the axonal diameter, undulation, curvature and so on. Any sensitivity to the diameter or curvature of axon, will change this power law. Obtaining an exponent of −1 for PTE and −1/2 for LTE could provide useful cross‐validation of the presence of stick‐like geometries in tissue. A complete analysis of the power‐law dependencies of the diffusion‐weighted signal at high b‐values has been performed. Only two forms of encoding result in a power‐law dependency, pure linear and pure PTE. The different exponents of these encodings could be used to provide independent validation of the presence of stick‐like geometries in vivo where, due to the slower decay, LTE is the most SNR efficient method. Deviation from the 1/√b power‐law in “stick‐like” geometries has been used recently to demonstrate sensitivity to the perpendicular diffusivity inside axons,^36^ which can ultimately be deployed to estimate the “effective MR radius” of the axons. In the same way, deviation from the power‐law for “pancake‐like” geometries would demonstrate a deviation from “thin‐film” geometry^32^ to “parallel plate” geometry, perhaps allowing the separation of the plates to be estimated (outside the scope of this work). We provided the first in vivo evidence for the power‐law relationship, 1/*b*, that has previously only been reported theoretically for PTE. The power‐law relationship in PTE was observed over the b‐value range of 7000 to 10 000 $s/{\mathrm{mm}}^{2}$. We also showed that a power‐law relationship only exists in an extremely limited set of conditions: 1. The substrate under investigation has “stick‐like” geometry and the b‐tensor shape is purely linear (one non‐zero eigenvalue) or purely planar (two non‐zero eigenvalues) or 2. The substrate under investigation has "pancake‐like" geometry and the b‐tensor shape is purely linear. A power law will not exist for any other axysymmetric b‐tensor. The effect of dot and spherical compartments on the signal decay was also investigated. The results show that LTE is not sensitive to small contributions of dot and spherical compartment. In contrast, the exponent in PTE shows sensitivity to the presence of other such compartments.

## APPENDIX A PLANAR TENSOR ENCODING

In PTE, $b_{\Delta }\;=\;-1/2$ and $S_{\mathrm{ic}}$ has the following form:

(A1) $$S_{\mathrm{ic}}^{\mathrm{PTE}}\left(b\right)\;=\;\frac{\sqrt{\pi }e^{\frac{-b{D_{a}}^{\left|\right|}}{2}}\;\text{erfi}\left(\sqrt{b{D_{a}}^{\left|\right|}/2}\right)}{2\sqrt{b{D_{a}}^{\left|\right|}/2}}$$

Asymptotic expansion of erfi(*x*) is as follows:

(A2) $$\text{erfi}\left(x\right)\;=\;\frac{e^{x^{2}}}{x\sqrt{\pi }}\sum_{k\;=\;0}^{\infty }\frac{(2k-1)!!}{{\left(2x^{2}\right)}^{k}}$$

where *x* → ∞ and (−1)!! = 1.

$b{D_{a}}^{\left|\right|}\;\gg \;1$ for large *b*, therefore we have:

(A3) $$S_{\mathrm{ic}}^{\mathrm{PTE}}\left(b\right)\approx \frac{1}{b{D_{a}}^{\left|\right|}}\sum_{k\;=\;0}^{N}\frac{(2k-1)!!}{{\left(b{D_{a}}^{\left|\right|}\right)}^{k}}$$

where *N* depends on the $b{D_{a}}^{\left|\right|}$ value (Figure 1 and Table 1).

(A4) $$S_{\mathrm{ic}}^{\mathrm{PTE}}\left(b\right)\;\approx \;\frac{1}{b{D_{a}}^{\left|\right|}}\left(1+\frac{1}{b{D_{a}}^{\left|\right|}}+\frac{3}{{\left(b{D_{a}}^{\left|\right|}\right)}^{2}}+\cdots \right)$$

## APPENDIX B SIGNAL ATTENUATION IN A CYLINDRICAL AND SPHERICAL PORE USING PTE

The signal attenuation of the impermeable cylinders^41^ using PTE is generated using the following equation^42^:

(B1) $$S_{\mathrm{cyl}}\;=\;{S_{\mathrm{cyl}}}^{\|}S_{\mathrm{cyl}}^{⊥}$$

(B2) $${S_{\mathrm{cyl}}}^{\|}\;=\;e^{-\frac{b}{2}{D_{a}}^{\|}\left(1-{\left(g.n\right)}^{2}\right)}$$

(B3) $$\ln\left(S_{\mathrm{cyl}}^{⊥}\right)\;=\;-\frac{2\gamma^{2}G^{2}\left(1+{\left(g.n\right)}^{2}\right)R^{6}}{{\left({D_{a}}^{\|}\right)}^{2}}\sum_{n\;=\;1}^{\infty }\frac{A_{n}}{\alpha_{n}^{6}\left(\alpha_{n}^{2}-1\right)}$$

where $\alpha_{n}$ is the root of the derivatives of the first order Bessel function $J_{1}^{\prime }\left(\alpha_{n}\right)\;=\;0$ and

(B4) $$A_{n}\;=\;\frac{2\alpha_{n}^{2}{D_{a}}^{\|}\delta }{R^{2}}-2+2L_{n}\left(\delta \right)-L_{n}\left(\Delta -\delta \right)+2L_{n}\left(\Delta \right)-L_{n}\left(\Delta +\delta \right)$$

and

(B5) $$L_{n}\left(t\right)\;=\;e^{-\frac{\alpha_{n}^{2}{D_{a}}^{\|}t}{R^{2}}}$$

$S_{\mathrm{Sph}}$ is the restricted diffusion in spherical pore using PTE^42^:

(B6) $$ln\;S_{\mathrm{sph}}^{\mathrm{PTE}}\;=\;-\frac{2\gamma^{2}G^{2}R_{s}^{6}}{D^{2}}\sum_{n\;=\;1}^{\infty }\frac{2A_{n}}{\beta_{n}^{6}\left(\beta_{n}^{2}-2\right)}$$

where $\beta_{n}$ is the root of the derivatives of the first order spherical Bessel function $j_{1}^{\prime }\left(\beta_{n}\right)\;=\;0$. For the diffusivity of the water molecules inside the spherical pore (*D*), we use a constant value of $1700\;\mu m^{2}/s$.

## APPENDIX C SNR AND ERROR

Let us assume that a real signal *S* follows a Rician distribution with parameters *A* and *σ*

(C1) S∼R(A,σ)

with PDF^45^

(C2) $$p\left(x|A,\;\sigma \right)\;=\;\frac{x}{\sigma^{2}}\;e^{-\frac{x^{2}+A^{2}}{2\sigma^{2}}}\;I_{0}\left(\frac{\mathrm{Ax}}{\sigma^{2}}\right)\;u\left(x\right),$$

where *A* is the (absolute value) of the original signal (without noise) and $\sigma^{2}$ is the variance of the complex Gaussian noise. It can be seen as

(C3) $$S\;=\;\sqrt{{\left(A+N_{r}\left(0,\;\sigma \right)\right)}^{2}+N_{i}{\left(0,\;\sigma \right))}^{2}}.$$

The question in MRI of how low can we go with the signal (i.e., when do we reach the noise floor) will always depend on the application and on the estimator we are using. However, we can always consider a lower bound to the SNR related to the error of the measured signal.

To calculate an SNR *threshold* independent of the particular application, we can use two different definitions of error: (1) the Mean Square Error (MSE) or (2) the mean error (ME). We define the MSE as

(C4) $$\text{MSE}\;=\;E\left\{{\left(S-A\right)}^{2}\right\}$$

where *S* is the measured signal and *A* is the *original* signal. We use the mean value to assure that this error is a statistical property and not an isolated measure. The ME is alternatively defined as:

(C5) $$\text{ME}\;=\;E\left\{S-A\right\}.$$

For the SNR calculation, we will consider that the error committed is a percentage of the original signal (to make it signal dependent), that is,

(C6) $$\begin{array}{cc} E\left\{{\left(S-A\right)}^{2}\right\} & \;<\;\epsilon \cdot A^{2}\\ E\left\{{\left(\frac{S}{A}-1\right)}^{2}\right\} & \;<\;\epsilon . \end{array}$$

Alternatively, for the ME:

(C7) $$\begin{array}{cc} E\left\{S-A\right\} & \;<\;\epsilon \cdot A\\ E\left\{\frac{S}{A}-1\right\} & \;<\;\epsilon . \end{array}$$

Assuming a Rician distribution of parameters *A* and *σ*, the errors become:

The MSE:

(C8) $$E\left\{{\left(\frac{S}{A}-1\right)}^{2}\right\}\;=\;2+\frac{2}{{\text{SNR}}^{2}}-\sqrt{2\pi }\frac{1}{\text{SNR}}L_{1/2}\left(-\frac{{\text{SNR}}^{2}}{2}\right)$$

The ME:

(C9) $$E\left\{\frac{S}{A}-1\right\}\;=\;\sqrt{\frac{\pi }{2}}\frac{1}{\text{SNR}}L_{1/2}\left(-\frac{{\text{SNR}}^{2}}{2}\right)-1.$$

For the sake of simplicity, in this paper, we will consider ME as an error measure, since MSE is more restrictive. The relation between ME and SNR for different errors can be seen in Table C1.

**Table C1:** SNR values for different errors for the Rician model

Error	<0.005*A*	<0.05*A*	0.1*A*	0.2*A*	<0.5*A*
ME	SNR > 10	SNR > 3.21	SNR > 2.30	SNR > 1.67	SNR > 1.05
